# Response to comments on my paper on whole body gestational donation

**DOI:** 10.1007/s11017-023-09646-y

**Published:** 2023-08-29

**Authors:** Anna Smajdor

**Affiliations:** University of Oslo, Blindern, Oslo, Norway

## To the Editor

In 2022, I published a paper in this journal as part of a Special Issue on controversial themes in bioethics [1]. My paper explored the possibility of extending existing organ donation frameworks to enable brain dead people to function as gestational surrogates for people who cannot—or prefer not to—gestate. I termed this ‘Whole Body Gestational Donation’ (WBGD). The point of my paper was primarily to show that WBGD would be consistent with practices that are currently accepted.

The commentaries I respond to here address various aspects of my argument. I cannot adequately address every point raised in this brief response. Instead, I have picked out a few salient issues to discuss in more detail. Before I discuss these, however, I would like to address a more general point about the justifiability of making arguments that call into question deeply held values or norms. The following passage from John Stuart Mill’s *On Liberty* explains my position on this:

Strange it is that men should admit the validity of the arguments for free discussion, but object to their being ‘pushed to an extreme’; not seeing that unless the reasons are good for an extreme case, they are not good for any case [2, p. 83].

The questions I raise in my paper are not frivolous, nor are they intended to provoke for the sake of provocation. They are posed in good faith, and I welcome those replies that engage with them as such.^1^

## Resource allocation

As several respondents noted, WBGD would have a significant impact on healthcare resources [4]. Giving one’s body for WBGD means the organs are not immediately available for other recipients, who arguably have greater need. Therefore, lives would be lost if WBGD were prioritised [5]. Even if organs could be donated after WBGD, they might no longer be of optimal quality. Thus, from a utility or justice perspective, it seems that WBGD is unacceptable [6].

However, my paper starts from the perspective of how things are, rather than from any theoretical position. There are many ways in which current organ donation systems fail to maximise utility (construed as saving lives). People can refuse to donate their organs or register dissent. They can choose to donate only specific organs. People may choose to leave their bodies to science rather than donate their organs.

The point is that we accept many constraints on utility in the context of healthcare. Whether WBGD could be construed as such a constraint requires further analysis. It is not sufficient simply to note that it would not maximise utility in terms of lives saved, at least for the purpose of my argument. An additional resource concern is the fact that WBGD would be extremely costly [5]. This could lead to WBGD being available only to the wealthy, and to a mismatch between the wealth and privilege of donors relative to prospective parents. However, my argument in relation to WBGD focusses primarily on issues related to its provision in *publicly* funded health services. In places where such services are absent or severely limited, all access to healthcare is dependent on finance, and tends to exacerbate existing inequalities. Therefore, there is nothing special about WBGD that marks it out as an unacceptable problem in these contexts.

With regard to WBGD’s cost-effectiveness within a publicly funded health service, this is a matter for economists to work out, rather than philosophers. However, there are some factors worth bearing in mind. Firstly, public health services commonly spend immense sums of money on medical procedures whose utility is open to question [7].

One way in which utility is reflected in health services is the reliance on the quality adjusted life year (QALY) measure. The efficacy of potential treatments is evaluated on their capacity to yield additional years of life to the patient treated, but in order to avoid simply saving lives at all costs, it also takes into account the *kind* of life being lived. QALYs are extremely controversial [8] but perhaps is the closest to mere utility that we can get [9].

Fertility treatments are problematic in the QALY system since they seem to trump all other considerations. If successful, fertility treatments generate an entire lifetime of QALYs, something that no other therapy can achieve [10]. I mention this here not to argue that we should therefore fund WBGD, but to demonstrate precisely the point made above: we apply utilitarian principles selectively, within certain predefined and frequently unquestioned parameters. Part of the purpose of my original paper was to expose and question these parameters.

A further consideration related to finance regards the money that health services currently spend on litigation for failures in obstetric care. In the UK, for example, these costs exceed those of all other areas of medical practice, and they are increasing [11].

Finally, many of these resource objections raised against WBGD could apply equally to the practice of organ donation and transplantation as a whole, especially in its early days. The expertise of the surgeon, the requirement for vastly expensive machinery, and the necessity for ongoing medication all make organ transplantation an extremely costly endeavour. Moreover, from a utilitarian perspective, transplantation is far from perfect. An organ ‘saves’ a life at significant cost: lifelong dependency on anti-rejection drugs that in turn impose an increased risk of diseases including cancer. And since transplanted organs have a limited lifespan, each recipient who survives long enough will have to undergo successive transplantations.

The point is that, even with traditional organ donation and transplantation, there are more cost-effective things we could do with our health resources. The fact that we choose to undertake transplants is representative of a deeper ideological value than mere utility.

## Pregnancy is not a disease

The beneficiaries of WBGD are those who cannot or prefer not to gestate. Some commentators thus construe WBGD as a kind of competition either between WBGD and conventional surrogacy [4], or between potential recipients of the donor’s organs and potential ‘recipients’ of the donor’s gestational capacity [6]. In this competition, it seems that WBGD loses, since on the one hand, surrogacy is preferable to WBGD in several ways [4]. And on the other, since pregnancy is not a disease: no-one *needs* WBGD in the way one needs a liver or kidney [6].

The starting point for Ber’s paper, and for mine, is that conventional surrogacy is morally problematic. For those who do not find this to be the case, there is no moral problem to address. While I cannot go into depth on the ethics of surrogacy here, it is worth reiterating a key point from my original paper: pregnancy is risky. In surrogacy, these risks are transferred to someone who may be harmed by them. In WBGD, they are transferred to someone who cannot be harmed by them. This gives WBGD a *prima facie* moral advantage over surrogacy, assuming that we wish to avoid harm to women.

It is true that pregnancy is not usually seen as a disease. A key point in my original paper was to show the degree to which we blithely accept many of the health risks of pregnancy—for women—which make their lives difficult, unpleasant, or in the worst case, which kill them. As I noted in my original paper, the lifetime risk of death from pregnancy is comparable to the risk of dying from measles, should one catch it [1]. Pregnancy has real and significant lifelong health impacts [12].

However, pregnancy is natural for women. We expect women to want children, and since pregnancy is the default route to biological motherhood (barring surrogacy), women are expected to undergo pregnancy with all its risks—gladly! Those who show hesitation or reluctance are pilloried in the media—and society generally—as being unfeminine or unfit for motherhood.

We castigate people who do not value their health enough to accept vaccinations, but at the same time, regard women who value their health enough to want to avoid pregnancy as being misguided, bizarre, or abhorrent. This is a clear example of cognitive dissonance. More than this, the belief that pregnancy does not merit the time, effort, and money that other comparable conditions do, is—I would suggest—reflective of a widespread form of misogyny.

Even so, this is not sufficient to prove that we should press ahead with WBGD. What it does mean is that we should take pregnancy seriously as a condition that is risky to one’s health, and which one might reasonably prefer to avoid while still desiring to become a mother.

## Sex/gender identity and trans donors

Some contributors object to my use of binary gender terms and find it problematic that I do not talk about trans people [6]. One of the reasons for this is the point made above. I am interested in the misogynistic attitudes that underpin society’s easy acceptance of women’s suffering in gestation and childbirth. These social attitudes are directed towards and experienced by people who are *identified by society* as women. This is particularly important when considering the historical trajectory of misogyny and sexism. Trans women experience oppression and discrimination too, but this is not my focus in this particular paper. My usage of ‘women’ is thus intended to capture a group which may include some trans and non-binary people, in terms of their own self-identification, but which is defined primarily in terms of how others perceive them.

In this context it is worth acknowledging that pregnancy is a longed-for aspiration for some people, wherever they fall on the sex/gender spectrum. However, the fact that some people wish for this experience should not override the interests of those who value their health and wellbeing above the experience of pregnancy itself, and for whom pregnancy is an obstacle in the path towards parenthood, rather than an end in itself.

## Consent

Some authors complain that I do not require explicit consent for WBGD in jurisdictions where implied consent is deemed sufficient for organ donation [4, 13]. By implication, these authors endorse the flimsy consent protocols currently operational in the organ donation context. My position on this is that, indeed, we should demand far more stringent consent protocols for ‘regular’ organ donation whether or not WBGD is a viable proposition. And certainly, WBGD should, on my view, if countenanced at all, be undertaken only with explicit, fully informed and freely given consent.

However, some commentators seem to suggest that even with optimal protocols, valid consent for WBGD would simply not be possible. WBGD would involve long term ventilation and involve many interventions, not all of which can be identified or predicted in advance [14]. Moreover, one cannot consent in advance to things that happen to one after one is dead. Therefore, valid consent for WBGD is simply not possible [15].

It is true that, once dead, one cannot give or withdraw consent, but this applies in any case where consent is given in advance for what happens after one’s death. Thus, if we insist that the ability to provide contemporaneous consent outweighs other considerations, not only would normal organ donation become impossible, but so would many other endeavours, such as the disposition of our property after death and our wishes concerning whether we should be buried or cremated. Indeed, if an ongoing capacity to give or withdraw consent is paramount, most surgery requiring general anaesthetic would also be ruled out.

Since we cannot consent after we are dead, the point—when considering posthumous interventions—is whether we can consent *before* we are dead. If not, then none of these posthumous activities are governed by consent at all. If we accept that consent to posthumous interventions before death can be valid in at least some of these cases, is there reason to think that WBGD is an exception?

The points about the exceptional duration and unpredictability of WBGD are not persuasive here. In current organ donation protocols, very few donors have an exhaustive knowledge of what is required. This, indeed, was one of the motivations for my original paper. We could, and should, do better. Aside from this, the business of harvesting organs is in itself unpredictable, since it relies on many complex clinical and social factors.

The long-term nature and unpredictable aspect of WBGD is comparable with donating one’s body for medical research, or for the use of medical students in their anatomy classes. In these contexts, the body will be maintained over a long period of time and used in ways that are not fully predictable. However, consent may be valid if potential donors are given as much information as possible about the likely trajectory of events, and if the inherent unpredictability is explained to them, and they choose to accept it.

## Conclusion

WBGD raises important questions about resource allocation, about the place of pregnancy in the hierarchy of medical need, and about the pressures that women experience as society’s gestators. In addition, WBGD leads us to think about the moral robustness of consent and to consider whether we could do better in terms of ensuring that today’s organ donors are treated ethically.

I have not discussed the medical feasibility of WBGD; nor have I explored WBGD from the perspective of the embryo/foetus/baby. These are important questions, but the former is best dealt with by clinical experts, and the latter requires more space than I can give to it here.

On the basis of this analysis, therefore, I conclude that there are still important reasons to think—critically—about WBGD.

## Footnotes

1 I do not engage with one of the responses—[3]—since it treats my paper as a policy recommendation, and thus misses the point of the original argument.
